# On Physically Unacceptable Numerical Solutions Yielding Strong Chaotic Signals

**DOI:** 10.3390/e24060769

**Published:** 2022-05-30

**Authors:** Wieslaw Marszalek

**Affiliations:** Department of Computer Science, Opole University of Technology, 45-758 Opole, Poland; w.marszalek@po.edu.pl

**Keywords:** numerical solvers, mathematical modeling, nonlinear dynamics, unphysical solutions, memristive circuits

## Abstract

Physically unacceptable chaotic numerical solutions of nonlinear circuits and systems are discussed in this paper. First, as an introduction, a simple example of a wrong choice of a numerical solver to deal with a second-order linear ordinary differential equation is presented. Then, the main result follows with the analysis of an ill-designed numerical approach to solve and analyze a particular nonlinear memristive circuit. The obtained trajectory of the numerical solution is unphysical (not acceptable), as it violates the presence of an invariant plane in the continuous systems. Such a poor outcome is then turned around, as we look at the unphysical numerical solution as a source of strong chaotic sequences. The 0–1 test for chaos and bifurcation diagrams are applied to prove that the unacceptable (from the continuous system point of view) numerical solutions are, in fact, useful chaotic sequences with possible applications in cryptography and the secure transmission of data.

## 1. Introduction

The modeling and analysis of nonlinear dynamical systems quite often require applications of various numerical solvers to obtain insights into complicated phenomena and their properties. Our world is nonlinear, complex and difficult to be understood, no matter if we deal with the nano ($10^{-9}$) or giga ($10^{9}$) scales of units. For example, the size of the COVID-19 virus is about 125 nanometers, while the new 5G communication applications use spectrum bands of frequencies from 6 to 100 GHz and beyond.

Mathematical modeling of nonlinear phenomena is based on using appropriate quantities and laws that the quantities obey. When many quantities interact with each other or are influenced by an unexpected turn of events, usually not well-understood and uncertain physically unacceptable outcomes may be generated, as illustrated by certain examples in COVID-19 analysis [1], climate predication [2] and economy modeling [3].

When a continuous model of a nonlinear event or phenomenon is constructed, it may already be flawed and inadequate in describing the underlying dynamical process. Bad measurement experiments or faulty measurement tools may further amplify the issue [4]. Thus, solving and analyzing such a model by a well-designed algorithm or solver may be of little to no value. On the other hand, when the continuous model is adequate and designed with precision, solving and analyzing it with an inappropriate tool (for example a poorly selected numerical solver or a solver with poorly chosen parameters) may also give problematic or completely unacceptable results. Both the above cases have been reported in the literature, see for example [5,6].

In this paper, we deal with the later problem of the first type described above when some incorrectly chosen numerical solvers or their chosen parameters are applied to the properly built continuous models. First, to introduce the topic (Section 2.1), we briefly present the known result in which the chosen numerical solver cannot be applied to a relatively simple linear system regardless of the chosen integration step size.

Then, the wrong computations discussed in the Section 2.2 are due to the incorrect choice (too large) of the integration step-size. The obtained solution is unphysical, as it violates the existence of the invariant plane of the continuous model (nonlinear system of ordinary differential Equations (ODEs)). Therefore, the obtained numerical solution for the integration step size of $10^{-2}$ cannot be considered as an acceptable physical solution of the continuous system. A physical and acceptable solution of the same continuous system is also presented in the Section 2.2. Then, in Section 3, we look at the former nonphysical numerical solution from a completely different point—namely, extracting strong chaotic signals.

Such chaotic signals may be of great importance to those who use chaos in, for example, the secure transmission of data, secret coding, or designing random number generators based on chaotic signals. Thus, our results in Section 3, to a large extent, illustrate the known proverb that, in some cases, one person’s trash (represented in this paper by the unphysical and not acceptable solution of a continuous system) is another’s gold (represented by a strong chaotic signal). This means that what one person considers worthless could be highly prized or valued by someone else. The main novel results in this paper are presented in the Section 2.2 and Section 3. Finally, Section 4 provides a short conclusion.

## 2. When the Numerical Solvers or Their Parameters Are Not Chosen Properly

It is well-known that care must be exercised when applying numerical solvers to continuous dynamical systems, such as those described by ODEs. Some solvers are completely useless for a particular system of ODEs, no matter what parameters are selected, others lead to wrong, physically unacceptable solutions for certain sets of parameters, while the same solvers result in correct solutions when the parameters are chosen properly. The example below (Section 2.1) illustrates the first (useless) approach, while the second example (Section 2.2) shows two completely different outcomes (incorrect and correct ones) for the same solver but with different sets of chosen parameters. The main goal of this subsection is to comment on a solution reported in the literature [7] that is not acceptable as it violates the presence of an invariant set of solutions and therefore must be rejected in nonlinear circuit analysis.

### 2.1. A Simple Case: Implicit and Explicit Euler Methods Do Not Work at All

It is well-known that the solution of $x^{\prime }=-y$, $y^{\prime }=x$ with the ${}^{\prime }\equiv d/dt$ and initial conditions x(0)=1 and y(0)=0 forms the unit circle in the y−x plane. When the explicit Euler method with the step size h>0 is used, we obtain the discrete model $x_{n+1}=x_{n}-hy_{n}$, $y_{n+1}=hx_{n}+y_{n}$. This discrete set yields $x_{n+1}^{2}=x_{n}^{2}-2hx_{n}y_{n}+h^{2}y_{n}^{2}$ and $y_{n+1}^{2}=h^{2}x_{n}^{2}+2hx_{n}y_{n}+y_{n}^{2}$. Denoting $r_{n+1}\equiv \sqrt{x_{n+1}^{2}+y_{n+1}^{2}}$, we have $r_{n+1}=\sqrt{x_{n}^{2}+h^{2}y_{n}^{2}+h^{2}x_{n}^{2}+y_{n}^{2}}=\sqrt{1+h^{2}}r_{n}$, which is an out-growing spiral in the y−x plane.

The obtained solution $\left(x_{n},y_{n}\right)$, n=0,1,⋯, is unacceptable as an approximate solution of the continuous system for all values of *h*, as, for n→∞, we obtain a spiral with increasing $r_{n}\to \infty$. Furthermore, the implicit Euler method (1-stage implicit Runge–Kutta method) applied to the same continuous problem yields $x_{n+1}=x_{n}-hy_{n+1}$, $y_{n+1}=y_{n}+hx_{n+1}$, and, after a simple manipulation, we arrive at $r_{n+1}=r_{n}/\sqrt{1+h^{2}}$, which is again unacceptable as a solution of the continuous problem, as, with n→∞, we obtain a spiral with decreasing $r_{n}\to 0$.

In both of the above methods, the numerical result is not a precise spiral due to the float-point arithmetic. The effect of round-off errors (important in other numerical problems [5,8]) is not even taken into consideration when rejecting the use of the above Euler methods.

### 2.2. Solving a Nonlinear ODE System Yielding a Wrong Outcome

Solving nonlinear systems of ODEs by numerical solvers is a more complex task as we usually do not know right away whether the obtain numerical solution approximates the continuous problem to an acceptable degree of correctness. In fact, the numerical solution may look initially acceptable to the *naked eye*; however, a deeper analysis of that numerical solution quite often results in its rejection as being flawed, physically unacceptable or violating certain properties of the nonlinear continuous ODE system. If such a deeper analysis is absent, and the numerical result is conveyed without any scrutiny, then we may observe an outcome similar to that presented in [7]—a published record with a flawed result that was not detected in the review process. Here are the facts.

**Figure 1:**
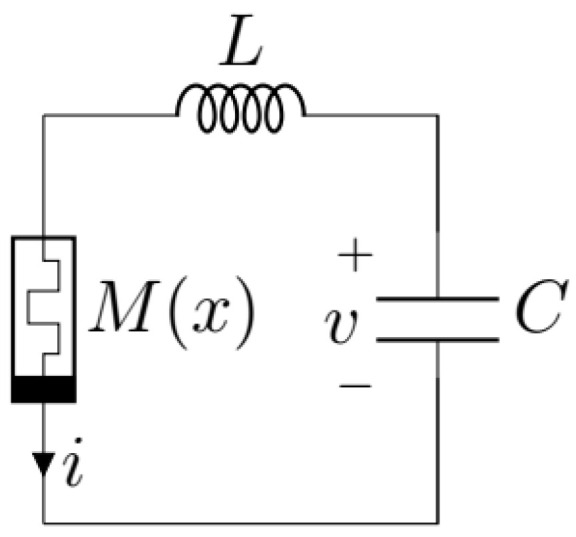
Memristive circuit.

A relatively simple three-element circuit (see Figure 1) with a series connection of a linear passive inductor *L*, linear passive capacitor *C* and a nonlinear active current-controlled *memristive device* with memristance M(x), with *x* being the memristor’s state variable, was considered in [7], resulting (after choosing a special form of M(x)) in the following model (see (69) in [7]).

(1) $$\begin{array}{ccc} x^{\prime } & = & \left(\lambda -1)x(y+\beta \right)\\ y^{\prime } & = & -\lambda z+\left(\lambda -1\right)\alpha \left(x^{2}-\gamma \right)y\\ z^{\prime } & = & \lambda y \end{array}$$

where λ=ω/(1+ω), $\omega =1/\sqrt{LC}$ and α, β and γ are positive constant parameters describing the memristive device. The variables *x*, *y* and *z* are the state variables of the memristor, scaled loop current *i*(y≡i/ω) and scaled voltage across the capacitor (z≡−Cv), respectively. It was assumed that the memristance $M\left(x\right)=\alpha L\left(x^{2}-\gamma \right)$ in the circuit in Figure 1.

**Figure 2:**
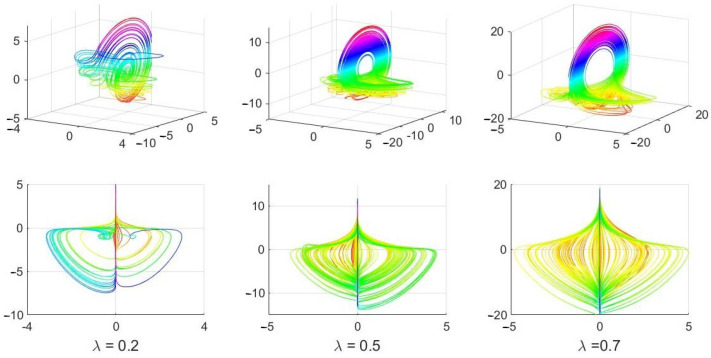
Solution of (1) obtained with *ode45* with dt=0.05, $relerr=abserr=10^{-2}$, [x(0),y(0),z(0)]=[0.5,0.5,0.5] and 0≤t≤8000 for three values of λ and constant α=β=1, γ=0.1. The first row shows the 3D solutions with the *colormap(hsv)* following the values of z(t) (vertical axis), while the second row shows 2D projections of the 3D solutions from the first row onto the x–y plane with the x-axis horizontal. All the solutions are drawn for 6500≤t≤8000.

The bifurcation of trajectories of (1) for fixed values of α=1, β=1, γ=0.1 and several values of 0≤λ≤1 are presented in Figure 36 in [7]. Although it is unclear what numerical solver and what parameters the authors of [7] used in their computation of the solutions in Figure 36 (the information is not provided in the published paper), one can quite easily obtain those solutions by using the popular *ode45* solver. Choosing dt=0.05, $relerr=abserr=10^{-2}$, initial conditions [0.5,0.5,0.5] (the same as in [7]) and 0≤t≤8000, yields practically the same solution figures. See Figure 2 below and compare the obtained solutions for the three selected values of λ with those in Figure 36 in [7].

The above described solutions (Figure 36 in [7] and Figure 1 in this paper) are not the solutions of the continuous system (69) in [7], which is (1) above.

*An explanation:* System (1) is such that the plane x=0 is invariant in the 3D space (x,y,z), and no trajectory originating with any positive initial value x(0)>0 can cross the plane x=0. In other words, no solution of (1) originating with the initial condition [x(0),y(0),z(0)] with x(0)>0 can reach the value $x(t^{*})=0$ for $t^{*}>0$ and proceed further to have x(t)<0. If the initial condition x(0) is positive, as it was assumed in [7] to create the solutions in Figure 36, and then the solution must be such that x(t)>0 for all values of t>0. The same is true if the initial condition for variable x(t) is negative: the solution must be such that x(t)<0 for all t>0.

Therefore, no crossing of the plane x=0 is possible. The plane x=0 is invariant, as, for x=0, we obtain dx/dt=0 from the first equation in (1), yielding x(t)=0 for all *t*. See also [9]. The trajectories in Figure 36 in [7] for 0<λ<1 are all incorrect, and they do not represent solutions of the nonlinear continuous system (69) in that paper.

**Figure 3:**
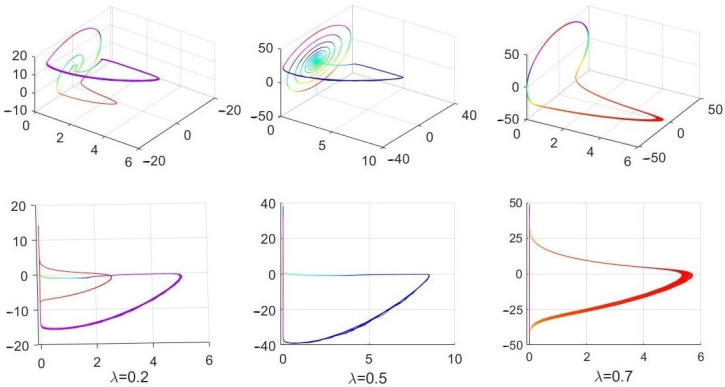
Solution of (1) obtained with $relerr=abserr=10^{-7}$. All other parameters are as in Figure 2. Notice that no solution trajectory exists for x(t)≤0 as the invariant plane x=0 cannot be entered into for any physically accepted solution of (1). Notice also the significant difference in the maximum and minimum values of the variables y(t) and z(t) between the solutions in Figure 2 and Figure 3 for λ=0.2 and 0.5 and to a lesser degree for λ=0.7.

When one reduces the *abserr* and *relerr* from $10^{-2}$ to $10^{-7}$, while keeping all other parameters unchanged, very different solutions of (69) in [7] are obtained by using the same solver. Those solutions are shown in Figure 3. The solutions do not reach (or further cross) the x=0 plane, are physically accepted and differ significantly from those unacceptable and incorrect solutions in Figure 36 in [7] (and in Figure 2 above).

## 3. Physically Unacceptable Solutions Yield Strong Chaotic Signals

While the unacceptable solutions of the nonlinear memristive circuit described in the previous section were obtained with relatively large values of *abserr* and *relerr*, one may attempt to look at those solutions from a completely different angle: the angle of chaos. Are the solutions in Figure 2 chaotic? Is the nonlinear circuit described by (1) chaotic? Would it be possible for a nonchaotic continuous circuit to yield good quality chaotic numerical solutions? Such seemingly contradictory statements are not out of the question, as we show below. If the answer to the later question is *yes*, what tools can be employed to provide an affirmative answer to such a question?

In this section, we address such issues and use the well-known tools to analyze the physically unacceptable solutions from the chaotic point of view. The method is to test the obtained solutions via the 0–1 test for chaos [10,11], which gained significant popularity in the analysis of chaotic signals in the last two decades [12,13,14,15,16,17]. For chaotic signals, one obtains the value of *K* (one possible result of the 0–1 test) close to 1, while any value close to 0 indicates a periodic signal. In this section, the physically unacceptable solutions of (1) are examined with the 0–1 test for chaos, and, in addition to the *K* values, other test results are also presented, e.g., p−q graphs [10].

One of the fundamental issues in a proper choice of the 0–1 test’s parameters is to prevent the *oversampling* phenomenon in the test [6,16]. This issue is related to the fact that the sequence of *c* numbers in the test is chosen randomly from the interval (0,π) and those numbers act as identifiers of the discrete frequencies present in the tested signals. Thus, typically, before the 0–1 test is applied to any signal, one should know the maximum discrete frequency $f_{max}$ in that signal. Then, based of that maximum frequency, a special parameter of integer value, say *T*, is chosen according to the rules presented in [6]. Then, every *T*th sample of the tested signal is selected and used to form a new sequence of values that is fed into the 0–1 test. Details and examples of such an approach are presented in [16]. A brief summary of the test is also provided in [18,19].

**Figure 4:**
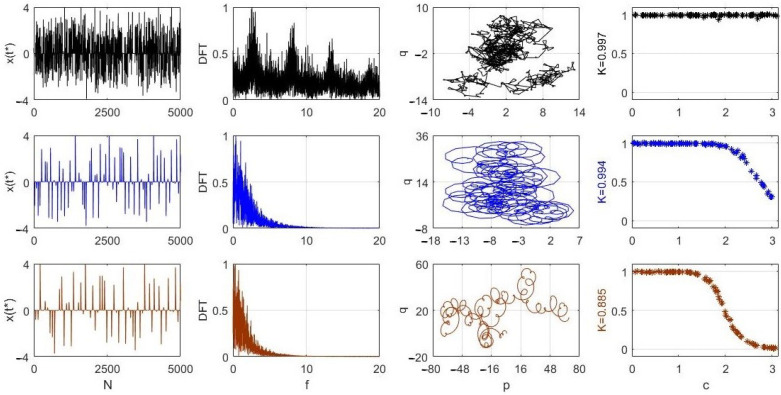
The 0–1 test result for the solution x(t) of (1) with λ=0.5, α=1, β=1, γ=0.1 and three values of *T*, namely 32, 4 and 3 for the cases in the first, second and third rows, respectively. Other details are given in the text.

**Figure 5:**
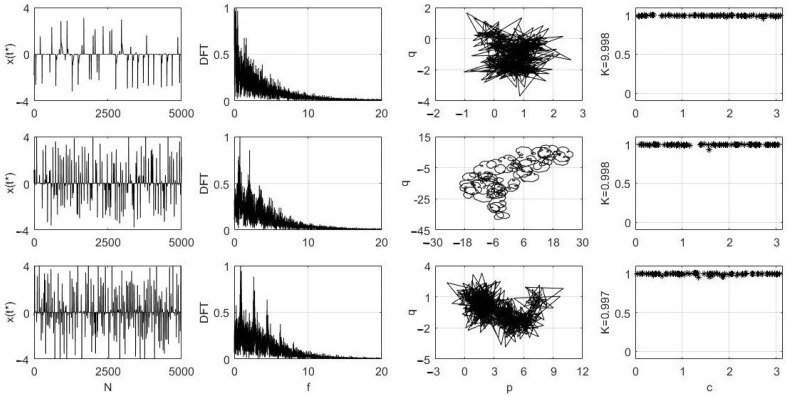
The 0–1 test result for the solution x(t) of Figure 2 for λ=0.2 (first row), λ=0.5 (second row) and λ=0.7 (third row). The T=8 for all three cases. Other details are given in the text.

The 0–1 test for chaos has been applied to the unphysical numerical solutions presented in Figure 2, and the results are shown in Figure 4 and Figure 5. First, since the solutions in Figure 2 were obtained with the integration step dt=0.05 and 0≤t≤8000, from the 160,000 values of x(t), we selected the discrete sequences backwards from the last (160,000th) one, by choosing every T=32, T=4 and T=3 solution samples of x(t) for the results presented in the first, second and third rows in Figure 4, respectively. In all three cases, each of the new sequences were restricted to 5000 values (see the first column in Figure 4).

This is due to the fact that, with T=32, the maximum length is 160,000/32 = 5000. The positive and negative spikes of *x* in each of the three cases in the first column indicate the trajectories crossing the invariant plane x=0 as described earlier in Section 3. The Discrete Fourier Transforms, or DFTs, for each sequence are shown in the second column in Figure 4. Next, the (p,q) variables computed in the 0–1 test (see [6]) are shown in the p−q plane in the third column. Finally, the most important results for each of the three sequences are shown in the fourth column in Figure 4.

For each of the three sequences, 100 values of *c*, 0<c<π (recommendation in [11]) were randomly selected, and for each of those 100 values, the corresponding $K_{c}$ values from the test were computed [6]. Then, the final value $K=median\{K_{c}\}$ was calculated as shown to the left of each of the three graphs in the fourth column. The obtained *K* values are very close to 1, strongly indicating that the analyzed three sequences are chaotic. For more details about the one-to-one correspondence of the DFT graphs in the second column and the $K_{c}$ graphs in the fourth column, see [6]. Furthermore, the p−q graphs in the third column show *Brownian-like* motion in each case, as they should in the case of chaotic signals [10].

Figure 5 shows similar results obtained for three values of λ (the same as in Figure 2). The new sequences were created by using T=8. Again, those sequences are chaotic, as the $K_{c}$ graphs and *K* values indicate in the fourth column in Figure 5.

**Figure 6:**
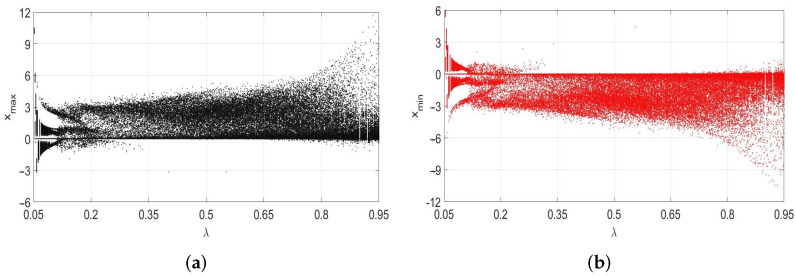
Bifurcation diagrams identifying the local maximum (**a**) and local minimum (**b**) values of *x* for 0.05≤λ≤0.95, 4000≤t≤8000, initial conditions [0.5,0.5,0.5], α=1, β=1, γ=0.1 and dt=0.05. The first half, 0≤t<4000, of the time interval is ignored when creating the above diagrams.

Figure 6 shows the bifurcation diagrams for the local maximum values of x(t) and local minimum values of x(t), both obtained by using x(0)>0. Notice the almost symmetrical bifurcation diagrams with respect to x=0 for the maximum and minimum values of *x*—that is, the diagram with $x_{min}$ is a reflection (vertical) of the diagram with $x_{max}$ with respect to x=0. Identical bifurcation diagrams for the local maximum and local minimum values of *x* are obtained if x(0)<0 is used. Figure 6 also confirms the spiking nature of the solution x(t) observed in the first columns in Figure 4 and Figure 5.

That is, for most values of the bifurcating parameter λ, the local maximum values of *x* are positive, with the exception of λs from 0.05 to approximately 0.25, and no local maximum values are negative for λ>0.25. The same is true with the local minimum values of *x* in Figure 6—they are all negative for the values of λ from 0.25 to 0.95, and no positive local minimum values occur in that interval of λ.

## 4. Conclusions

Section 3 with examples of chaotic sequences obtained from physically unacceptable solutions of (1), and equivalently of (69) in [7], shows that sometimes a numerical solution of a system of ODEs is not acceptable—that is, it does not represent a solution of the continuous system’ yet, as the 0–1 test of chaos and the bifurcation diagrams indicate, may result in valuable strong chaotic sequences. Such chaotic sequences can find application in chaos-based engineering designs. There is, at the end, a positive outcome of the analysis of the memristive circuit and its model (1).

One can expect similar positive outcomes in other nonlinear dynamical circuits and systems, with the computational chaos reported in [20,21], or in other circuits in which both passive and active elements are present. Questions and issues related to those presented above are also analyzed in [22], where questions of the reliability of the chaotic solutions are raised. Furthermore, in [23], certain examples of systems with simple analytical solutions are presented, such that the chaotic orbits do not track them.

Certain memristive circuits [24,25,26,27,28,29,30,31,32], with elements having pinched hysteretic current–voltage characteristics, are described not by ODEs but by DAEs (Differential-Algebraic Equations), and such mathematical models usually have singularities (typically behaving as impasse points) [33]. Crossing such impasse points by solution trajectories is, in principle, prohibited. However, in an *ill-designed* numerical problem, such a crossing is expected to be possible, as with the cases described in this paper.

## Data Availability

The data and figures generated during the current study are included and discussed in the paper.
